# The Extrinsic Incubation Period of Zika Virus in Florida Mosquitoes *Aedes aegypti* and *Ae. albopictus*

**DOI:** 10.3390/pathogens10101252

**Published:** 2021-09-28

**Authors:** Rebecca A. Zimler, Barry W. Alto

**Affiliations:** Florida Medical Entomology Laboratory, Entomology and Nematology Department, Institute of Food and Agricultural Sciences, University of Florida, 200 9th St. S.E., Vero Beach, FL 32962, USA; razimler@ufl.edu

**Keywords:** arbovirus mosquito vectors, emerging pathogen, Zika virus infection

## Abstract

The Asian genotype of Zika virus (ZIKV) emerged in Brazil in 2015 and subsequently spread throughout the Americas. In July 2016, Florida experienced its first locally acquired ZIKV infection in the continental U.S. Concerns about health risks from ZIKV infection have increased the need to investigate the interactions between potential mosquito vectors and ZIKV. The time it takes for an arbovirus to propagate within a mosquito, and become transmissible, is the extrinsic incubation period (EIP). The EIP for potential mosquito vectors in Florida is unknown. To address this gap in the understanding of ZIKV epidemiology, Florida *Aedes aegypti* (L.) and *Ae. albopictus* (Skuse) were orally exposed to ZIKV infected blood meals and fully engorged mosquitoes were held at a constant temperature of 28 °C through the duration of the experiment. Saliva expectorates were collected from cohorts of mosquitoes and tested for the presence of ZIKV at three-day intervals over a period of 24 days to allow for an evaluation of the EIP of the emergent Asian lineage of ZIKV. High rates of infected bodies in *Ae. albopictus* (75–94%) and *Ae. aegypti* (68–86%) were observed throughout the incubation period, which did not differ by species. Higher rates of disseminated infection were observed later during the incubation period but did not differ between species. We calculated the 50% EIP to be shorter in *Ae. albopictus* than *Ae. aegypti* (16.2 and 18.2 days post infection, respectively). The competence for ZIKV observed in both species may contribute to high rates of ZIKV transmission in Florida populations.

## 1. Introduction

Zika virus (ZIKV) is a primarily mosquito-borne *Flavivirus* first identified in Uganda in 1947 [1]. ZIKV infection in humans is often asymptomatic (approximately 80%), although infection can cause acute febrile illness with clinical presentations including malaise, fever, maculopapular rash, headache, and conjunctivitis [2]. Severe illness associated with ZIKV infection, such as microcephaly and Guillain Barré syndrome, has caused global concern for the disease and sparked an urgent need for the understanding of ZIKV epidemiology. The first large outbreaks of ZIKV in humans occurred in the Pacific on the island of Yap, in the Federated States of Micronesia in 2007 and French Polynesia in 2013. In 2015, Zika infection spread throughout Brazil and subsequently the Americas [3,4]. The first evidence of local transmission of ZIKV in Florida occurred in Miami-Dade County in 2016. Additionally, sporadic cases of locally acquired Zika infection and imported cases in Florida were identified in Pinellas, Broward, and Palm Beach counties [5].

Zika virus is typically spread through the bite of an infected mosquito but direct transmission may also occur through sexual contact, in utero, perinatal, and blood transfusion in asymptomatic donors [2]. *Aedes aegypti* (L.) is considered the primary vector in the urban cycle of ZIKV in the Americas [6,7] and viral isolates and laboratory vector competence studies have implicated the invasive *Ae. albopictus* (Skuse) as a secondary vector [6,7,8,9,10]. In some cases, *Ae. albopictus* alone has been responsible for local transmission and outbreaks of arboviruses (dengue and chikungunya viruses) [8,9,10,11,12,13,14,15]. In Florida, *Ae. aegypti* and *Ae. albopictus* were potential vectors of ZIKV during the Miami-Dade 2016–2017 outbreak [2]. Although local transmission of ZIKV in Florida has ceased, introduction of the virus from travel-related cases continues to be reported. Local transmission in the U.S. is a major public health risk, especially for Florida where the main mosquito vectors reside and are abundant throughout much of the year and there is a high potential for virus re-introduction from human travel [5].

*Aedes aegypti* and *Ae. albopictus* transmit some of the most significant arboviruses affecting human health. The reason for their primary role in transmitting emergent arboviruses largely stems from anthropogenic tendencies. Both mosquito species are considered invasive and can live near human habitats which afford them numerous opportunities to achieve enhanced vectorial capacity (*C*), an index that incorporates intrinsic and extrinsic factors to estimate the risk of disease transmission (the number of secondary infections expected to occur from the introduction of a single infection in a naïve population). 

Vectorial capacity is calculated as below:

*C* = [*ma*^2^ (I*T)*p^n^*]/−*ln*(*p*) (1)

where *m* is vector density in relation to the host, *a* is the probability that a vector feeds on a host in one day, *p* is the probability that a vector survives one day, *n* is the duration of the extrinsic incubation period (EIP) in days, *I* (infection rate) *T (transmission rate) is equivalent to vector competence (*b*). The duration of the vector’s life in days after surviving the EIP is calculated as 1/−*ln*(*p*). This equation shows that host feeding (*a*), vector longevity (*p*) and EIP (*n*) influences *C* strongly as a square or exponent function, and vector competence is a weaker contributor in determining *C*. In short, EIP is one of the most influential determinants of a mosquito’s ability to transmit a pathogen, and its contribution can be modified depending on the relationship with vector longevity [16].

Virus infectivity for mosquitoes, incorporated into *C* as *b*, is a weaker contributor compared to the time to disseminated infection and viral infection of the salivary glands that would impact the duration of the EIP, which would influence *C* as *n* [16]. The EIP_50_ is calculated as the time in days from imbibing a pathogen until 50% of mosquitoes have the ability to vector the pathogen. The EIP_50_ is based on the functional fit to multiple time points in the growth phase of the process of vector competence and can be a better measure than single-time-point sampling for characterization of virus efficiency within the vector. The EIP of ZIKV has an average range of 3–14 days, although under some conditions, the EIP of ZIKV has been found to take as long as 21 days post infection (dpi) [17,18,19]. The wide range in EIP of ZIKV can be influenced by intrinsic (mosquito and viral genetics) and extrinsic factors (temperature) [20,21,22]. Therefore, it is important to understand the EIP of ZIKV within and between vector populations to appropriately analyze vectorial capacity under specific circumstances. 

The present study uses ZIKV from Puerto Rico to determine relative infection, disseminated infection, and transmission rates of *Ae. aegypti* and *Ae. albopictus* mosquitoes as well as the EIP. We make use of local populations of *Ae. aegypti* and *Ae. albopictus* mosquitoes from Florida under temperatures representative of the daily temperature observed in central Florida during late summer/early fall to coincide with peak transmission and appraise local risks of its transmission and emergence [23].

## 2. Results

### 2.1. Susceptibility to Zika Virus Infection

Viral titrations of the infectious blood meal showed a final concentration of 7.5 Log_10_ PFUe/mL. Mosquito bodies were tested for the presence of ZIKV infection at eight time points post infection by qRT-PCR (446 mosquitoes; 359 *Ae. aegypti*, 87 *Ae. albopictus*). Logistic regression analysis showed no significant difference between species (df = 1, χ^2^ = 3.25, p = 0.0710), time (dpi) (df = 7, χ^2^ = 4.21, p = 0.7557), or their interaction (df = 7, *χ^2^* = 2.12, p = 0.9527). *Aedes aegypti* and *Ae. albopictus* females had similar susceptibility to infection during the time monitored after ingestion of Zika virus-infected blood (Table 1). The lack of significant interaction between mosquito species and time indicates that both species responded similarly to altered susceptibility to infection at the various time points tested. Infection rates were high during the entire incubation period ranging from 68–86% for *Ae. aegypti* and 75–94% for *Ae. albopictus* mosquitoes (Table 2).

### 2.2. Disseminated Infection

Legs of fully engorged individual mosquitoes were examined for disseminated infection. There was a significant effect of time (χ^2^ = 70.43, df = 7, p ≤ 0.0001). However, the species (χ^2^ = 2.94, df = 1, p = 0.1145) and species by time interaction were not significant (χ^2^ = 10.62, df = 7, p = 0.1558) (Table 1, Table 3). A follow-up test on the time effect using the Tukey-Kramer procedure showed significant differences (all p < 0.04) at 3 vs. 24 dpi, 3 vs. 21 dpi, 3 vs. 18 dpi, 3 vs. 15 dpi, 3 vs. 12 dpi, 3 vs. 9 dpi, 6 vs. 12 dpi, 6 vs. 15 dpi, 6 vs. 18 dpi, 6 vs. 21 dpi, 6 vs. 24 dpi, 9 vs. 21 dpi, and 9 vs. 24 dpi, with later time points having higher rates of disseminated infection (Table 3).

### 2.3. Saliva Infection 

The presence of ZIKV RNA in saliva was examined from individual mosquitoes that previously imbibed an infected blood meal at the start of the infection study. There were no significant effects of species (df = 1, χ^2^= 0.03, p = 0.8523). However, there was a significant effect of time (df = 7, χ^2^ = 19.98, p = 0.0056) and a species by time interaction (df = 7, χ^2^ = 14.59, p = 0.0416) (Table 1). A follow-up test on the time effect using the Tukey–Kramer procedure showed significant differences (all p < 0.02) at 3 vs. 15 dpi and 6 vs. 15 dpi, with later time points having higher rates of saliva infection (significant time effect, Table 3). Transmission rates were 0–50% and 0–45% for *Ae. albopictus* and *Ae. aegypti* (nonsignificant species effect), respectively. After adjusting for multiple comparisons, we did not detect significant differences in saliva infection between any of the treatment groups for the significant species by time interaction (Table 4). A follow-up test on the time effect using the Tukey-Kramer procedure showed significant differences (all *p* < 0.02) at 3 vs. 15 dpi and 6 vs. 15 dpi, with later time points having higher rates of saliva infection (Table 3). Transmission rates were 0–50% and 0–45% for *Ae. albopictus* and *Ae. aegypti*, respectively.

### 2.4. Extrinsic Incubation Period (EIP_50_)

The probit procedure by species was run to determine the probability values for the EIP_50._ The mean extrinsic incubation period was calculated as the time from ingestion of the ZIKV infectious blood meal until 50% of infected females were capable of transmission (present in expectorated saliva). The EIP_50_ values were found to be 16.2 dpi for *Ae. albopictus* and 18.2 dpi for *Ae. aegypti* (Table 5). 

### 2.5. Viral Load 

The mean body titers for *Ae. aegypti* and *Ae. albopictus* exposed to ZIKV and examined at 3, 6, 9, 12, 15, 18, 21, and 24 days later are reported in Table 6. Results from the ANOVA showed a significant difference between time (df = 7, χ^2^ = 25.11, p < 0.0001) and a lack of difference between the species (df = 1, χ^2^ = 0.01, p = 0.9746), and the species by time interaction (df = 7, χ^2^ = 1.18, p = 0.1966). The time effect showed viral titers in bodies were significantly higher on days 12, 15, 21, and 24 than on days 3, 6, 9, 18 (Table 6). The mean leg titers for *Ae. aegypti* and *Ae. albopictus* exposed to ZIKV and examined at 3, 6, 9, 12, 15, 18, 21, and 24 days post exposure are reported in Table 7. Results from the ANOVA showed a significant difference between species (df = 1, χ^2^ = 9.63, p = 0.0135), and time (df = 7, χ^2^ = 4.59, p = 0.0057) and a lack of difference between the species by time interaction (df = 7, χ^2^ = 1.67, p = 0.3780). Significantly higher viral loads in legs were observed in *Ae. aegypti* compared to *Ae. albopictus*. The time effect showed significantly higher viral titers in legs on days 18, 21, and 24 than 6 and 9 and significantly higher viral loads in legs on day 15 than day 9. 

The viral load was measured in saliva expectorates from mosquitoes that ingested ZIKV virus infected blood and were examined at 3, 6, 9, 12, 15, 18, 21, and 24 days later (Table 8). Results from the ANOVA show a lack of significant differences between the species (df = 1, χ^2^ = 2.37, p = 0.3310), time (df = 6, χ^2^ = 2.80, p = 0.3516), and the species by time interaction (df = 5, χ^2^ = 2.34, p = 0.4583).

## 3. Discussion

This study was designed to determine the EIP of an emergent genotype of ZIKV using local populations of *Ae. aegypti* and *Ae. albopictus* mosquitoes from Florida and provide an assessment of the relative vector competency for ZIKV by these mosquito species. Our evaluations of the EIP for *Ae. aegypti* and *Ae. albopictus* were performed under a temperature environment that approximates the daily temperature observed in central Florida during late summer and early fall. Both species responded similarly regarding susceptibility to infection and disseminated infection. The extrinsic incubation period was shorter for *Ae. albopictus* than *Ae. aegypti*, although we observed overlapping 95% Fiducial Limits (Table 5). The saliva sample size is low in *Ae. albopictus* and estimates of percent infection at any one time point are likely to be highly variable and inaccurate due to a low sample size which may account for the proportional difference observed.

Few studies have investigated the EIP of ZIKV in *Ae. aegypti*, primarily using long-standing colonies and testing only a few time points. Chouin-Carneiro et al. [6] investigated the incubation period of ZIKV from New Caledonia in Florida derived *Ae. aegypti* and *Ae. albopictus* sampled at three time points (4, 7, and 14 dpi). The results of the study concluded that the EIP for both species examined was longer than 7 days. This study performed by Chouin-Carneiro et al. used long-standing colonies of >F10 *Ae. aegypti* and F7 *Ae. albopictus*. Research has shown laboratory colonies maintained for several generations (F7–10) may not be representative of field populations. Vazeille et al. [24] showed that dengue-2 infection rates of *Ae. albopictus* were up to 4-fold different in recently collected versus older laboratory strains, suggesting that laboratory colonization alters susceptibility to infection. 

Species-specific variations in EIP have also been reported. For example, Hugo et al. [25] orally exposed established colonies of *Ae. aegypti* and *Ae. albopictus* from Australia to an infectious blood meal containing a Brazilian ZIKV strain. The results from the Hugo et al. study revealed a shorter EIP of 10 dpi in *Ae. aegypti* compared to the EIP of 14 dpi expressed in *Ae. albopictus* [24]. These results do not agree with the findings in our study, where *Ae. aegypti* displayed a longer EIP compared to *Ae. albopictus*. Similarly, a study examining the impact of temperature on the EIP of ZIKV in *Ae. aegypti* from California showed that EIP_50_ estimates were 5.1 days at 30 °C, 9.6 days at 26 °C, and 24.2 days at 21 °C [26]. The differences observed between EIP across studies could be attributable to the difference in incubating temperatures used, mosquito genetics, or viral strain used. As time from oral ZIKV infection progresses, the probability of transmission increases in *Ae. aegypti. Ae. albopictus* seems to have the opposite effect where the probability of transmission potential decreases over time. One plausible mechanism that would account for decline in transmission potential with the length of infection is virus modulation of the infection by the mosquito [27,28]. These differences in EIP_50_ may play a critical role in ZIKV transmission and could have implications during outbreaks where the two species coincide. Collectively, these observations suggest a range of EIPs for ZIKV within and between vector populations and underscores the importance of investigations of different mosquito species and geographic populations of the same species because of known genetic variation. For example, an infection study by Alto et al. [29] showed small-scale geographic variation in vector competence measurements for chikungunya virus in *Ae. aegypti* and *Ae. albopictus* in Florida [29]. However, their study did not measure EIP or identify the mechanism (e.g., genetic difference among mosquitoes or differences in the microbiome, virome, or immune activation). 

In order for ZIKV to infect the salivary glands and become a potential transmitter of the virus, ZIKV must first infect the midgut epithelium after an infectious bloodmeal, replicate in the midgut epithelium cells, pass through the basal lamina and escape the midgut barrier to replicate in other organs and tissues, infect the salivary glands, and lastly, escape into the lumen of the salivary gland in order for the mosquito to infect the host [29]. In our study, most mosquitoes of both species (68–81%) were infected by 3 days post exposure, and infection rates remained steady for the duration of the experiment. Chouin-Carneiro et al [6] observed similarly high infection rates in *Ae. aegypti* and *Ae. albopictus* from the USA (approximately 90% and 75%, respectively) examined 4 dpi. We observed higher rates of individuals with disseminated infection among Florida *Ae. aegypti* than *Ae. albopictus*, whereas Chouin-Carneiro et al. [6] determined no differences in the dissemination of ZIKV between the two species. However, the disseminated infection rates observed in the study by Chouin-Carneiro et al. [6] were approximately 20%, substantially lower than our study. The data in our study shows a trend for increased disseminated infection with time after mosquitoes imbibed ZIKV infected blood meal and the results from the logistic regression analysis confirm this trend showing a significant effect of time on disseminated infection. We observed a significant effect of the time factor on mean viral load in the bodies of *Ae. aegypti* and *Ae. albopictus*. Overall, the time effect showed body titers increased in line with time after ingestion of ZIKV infected blood. A similar relationship was seen in the viral load of disseminated infection. Additionally, significantly higher viral loads were found in the legs of *Ae. aegypti* compared to *Ae. albopictus*. Higher dissemination titers in *Ae. aegypti* compared to *Ae. albopictus* have been documented in other studies, and likely contributes to the increased transmission efficiency of *Ae. aegypti* [24]. Viral replication kinetics may be responsible for the differences in viral loads between the mosquito species observed in these studies. 

In the current study, saliva infection was very low following the first few days after imbibing infected blood. This observation differs from saliva infection with chikungunya virus using local populations of *Ae. aegypti* and *Ae. albopictus* from Florida showing relatively high saliva infection rates early during infection [29]. Differences in the progression of infection between Zika and chikungunya viruses in mosquitoes may be attributable to differences in viral replication strategies [28]. In addition to the EIP, duration of salivary infection has important ramifications for public health and control. In our study, infected saliva was detected in most individuals of *Ae. aegypti* between 18 and 24 dpi, and at 15 dpi in *Ae. albopictus*. These results differ slightly from other studies. Di Luca et al. [30] offered an infectious blood meal containing an Asian genotype of ZIKV to *Ae. albopictus* mosquito populations from Italy and a long-standing colony of *Ae. aegypti* from Mexico. Saliva was collected in capillary tubes at different time points after blood feeding. The virus was detected in the saliva of *Ae. aegypti* as early as 3 dpi and persisted until 21 dpi, peaking between 11–18 dpi. Infected saliva was only detected in *Ae. albopictus* at 11 and 14 dpi [30]. These findings suggest the *Ae. albopictus* from Italy have a shorter persistence of ZIKV in saliva compared to *Ae. aegypti* and *Ae. albopictus* from Florida. These extended salivary infection periods indicate a longer amount of time that these mosquitoes are potentially infectious to humans and may contribute to less effective control efforts.

EIP is one of the most influential parameters of vectorial capacity because EIP (*n*) and probability of daily survival (*p*) interact exponentially [18]. A vector must survive long enough for the completion of the EIP, and the probability of daily survival by a vector is principally dependent on environmental conditions [29]. This exponential interaction between EIP and daily survival is the reason that variation in EIP has the ability to influence vectorial capacity in a much greater manner compared to similar changes in other factors such as vector density or vector competence. A decrease in EIP by a single day can dramatically increase vectorial capacity when other factors remain constant. Our study analyzed the probability of transmission over time. We found as time from oral ZIKV infection progresses, the probability of transmission increases in *Ae. aegypti* and *Ae. albopictus* seem to have the opposite effect where the probability of transmission potential decreases over time (Table 2). For example, the EIP_25_, or the time from ingestion of ZIKV until 25% of infected females were capable of transmission_,_ for *Ae. albopictus* was almost 21 dpi compared to 5 dpi in *Ae. aegypti*. Studies evaluating EIP of ZIKV report variable results [25,30]. Utilizing various probabilities of EIP, such as EIP_25_ and EIP_50_, could help provide a better parameterization of EIP in vectorial capacity models for improved accuracy of prediction [31]. 

Our findings show that ZIKV was present in saliva expectorates during the entire incubation period, an amount of time that likely exceeds the lifespan of mosquitoes in nature. Other infection studies in *Ae. aegypti* and *Ae. albopictus* have shown a decline in transmission efficiency, perhaps attributable to virus modulation [27,28]. The persistence of the virus in the mosquito can contribute to high rates of ZIKV transmission, especially in vectors that display gonotrophic discordance. Gonotrophic discordance is an event in which multiple blood meals are ingested during a single gonotrophic cycle. This phenomenon provides the female with multiple chances to ingest and transmit a pathogen multiple times per gonotrophic cycle [32]. *Ae. aegypti* exhibit gonotrophic discordance and are highly anthropophilic meaning they strongly prefer to feed on human blood [33,34,35]. The anthropophilic nature may influence the likelihood of a vector feeding on an infected human host during an outbreak and thus, increases the likelihood of horizontal and vertical transmission of ZIKV [6]. This impact may be enhanced with an extended duration of viremia. 

The viral load of ZIKV-infected saliva (Table 8) was not found to be significantly different between the treatment factors examined and, in part, can be attributable to sample size. The minimum infectious dose for humans to become infected through the bite of a ZIKV-infected mosquito is unknown. However, nonhuman primate studies using rhesus macaques have been used as a model to study ZIKV transmission. Dudley et al [36] allowed ZIKV (Puerto Rico Strain) infected *Ae. aegypti* (Liverpool strain) to feed on rhesus macaques and were later examined for infection. The estimated infectious saliva from the mosquitoes was between 1.5–3.2 Log_10_ PFU/mL. The results from this study found that these low doses were high enough to infect the macaques. It is suggested that the infective dose could contribute to the variation in disease outcomes observed from ZIKV infection and that higher titers of infectious saliva from mosquitoes could result in a more rapid time of peak in viral loads [36]. 

The results from our study characterized ZIKV infection and EIP_50_ in *Ae. aegypti* and *Ae. albopictus.* We observed a shorter EIP_50_ in *Ae. albopictus* (16.2 dpi) compared to *Ae. aegypti* (18.2 dpi). The EIP of other arboviruses can be influenced by mosquito strain, temperature, and infectious dose [16,28,29,37]. Future studies should investigate the impact of these factors on EIP for ZIKV in Florida populations of mosquito vectors to help generate parameters that can be used in predicting the risk of ZIKV outbreaks in Florida. 

## 4. Materials and Methods

### 4.1. Mosquitoes 

Two recently collected mosquito vectors from Florida were tested for ZIKV competence and their relative EIPs. *Aedes aegypti* and *Ae. albopictus* were obtained from larvae collected from the field at a tire yard in Okeechobee, FL in June 2016. For routine mosquito colony maintenance, the parental generation larvae from field collections were reared under standardized conditions at 28 °C with a 15 h:9 h light:dark (L:D) cycle and approximately 80% relative humidity. The photoperiod approximates the longest day length (sunrise-sunset) naturally observed in summer in Florida. Pupae were collected from rearing containers and placed in sealed shell vials until eclosion. Newly emerged adults were identified to species and the adults were allowed to freely mate in Bugdorm insect rearing cages (30 cm^3^). Mosquitoes were supplied with a 10% sucrose solution on cotton wicks and provided defibrinated bovine blood (Hemostat Laboratories, Dixon, CA, USA) in hog casings heated to 37 °C. The F_2_ generation eggs from these Okeechobee populations were used in the infection study. *Aedes aegypti* and *Ae. albopictus* eggs were hatched using an electrically powered pump and an insulated vacuum container to hatch the eggs synchronously. Larvae were reared at a density of 200 larvae/L of water with the same photoperiod and temperature as indicated previously, and fed a diet of 1:1 brewer’s yeast:liver powder formula. Pupae were removed from larval pans and placed in water-filled cups inside Bugdorm cages for eclosion. Adult mosquitoes were provided with a 10% sucrose solution and water and held until adults were 7–8 days old.

### 4.2. Virus Isolate and Propagation

An Asian lineage of ZIKV from Puerto Rico (strain PRVABC59, GenBank: KU501215.1) was used to infect the mosquitoes. The virus was isolated from human serum during the outbreak in 2015 and obtained from the Centers for Disease Control and Prevention. We passaged ZIKV twice in Vero cells before use in the infection study. The ZIKV strain responsible for the 2015 outbreaks occurring in the Americas was purposely chosen for this study due to the importation risk to Florida. Zika virus used in the infection study was propagated in monolayers of Vero cells with M199 medium supplemented with 10% fetal bovine serum, 2% penicillin/streptomycin, and 0.2% mycostatin at 37 °C and 5% CO_2_ atmosphere. Confluent monolayers of Vero cells were inoculated with ZIKV at a multiplicity of infection of 0.1 plaque-forming units (PFU) per cell. After 1 h of incubation, 24 mL of complete media was added to the infected monolayers and incubated for 6 days prior to incorporating defibrinated bovine blood for the oral infection studies. 

### 4.3. Per os Infection of Mosquitoes 

Female mosquitoes were cold anesthetized at 4 °C and sorted into cohorts of 55 individuals, separated by species. The mosquito cohorts were placed into cylindrical cages (10 cm ht. × 10 cm top dia. × 7 cm bottom dia.) with mesh tops 24 h before per os infection. Mosquitoes were moved into an incubator maintained at 28 ± 1 °C with 15 h:9 h L:D cycle in the biosafety level 3 facility at the Florida Medical Entomology Laboratory. The temperature chosen is representative of daily temperatures observed in central Florida during late summer/early fall (National Oceanic Administration Association: http://cdo.ncdc.noaa.gov/ulcd/ULCD) when the risk of ZIKV transmission is the highest. 

Mosquitoes were sugar starved for 15 h prior to infectious blood meal feeding and deprived of water 1 h prior to feeding to increase feeding rates. Mosquitoes were offered a freshly propagated ZIKV-infected blood meal consisting of defibrinated bovine blood, adenosine triphosphate (ATP) at a 0.005 M concentration added as a phagostimulant, and ZIKV using a Hemotek membrane feeding system (Lancashire, United Kingdom) warmed to 37 °C. Viral titrations of the infected blood showed a final concentration of 7.5 Log_10_ PFUe/mL. After feeding for 1 h, mosquitoes were cold anesthetized for sorting. Fully engorged mosquitoes were sorted into groups of 50 females, transferred into cages by species and given 10% sucrose solution. Cohorts of mosquitoes were tested every 3 days at eight time points (3, 6, 9, 12, 15, 18, 21, and 24 days post exposure to infectious blood (dpi) for the transmission potential of ZIKV by tests of saliva expectorates for ZIKV RNA collected in capillary tubes with immersion oil as previously described [38]. Mosquito samples were stored at −80 °C until tested.

### 4.4. Processing Mosquitoes and Detection of Zika Virus 

Mosquito samples were thawed and individually dissected to remove legs from bodies. Mosquito bodies, legs, and saliva were tested separately to characterize susceptibility to infection, disseminated infection, and transmission potential, respectively. The denominator for calculations of vector competence measurements (bodies, legs, saliva) was the number of fully engorged mosquitoes. Individual body and leg samples were homogenized in centrifuge tubes with 1 mL of media and two steel BBs at 26 Hz for 3 min using a TissueLyser II (Qiagen, Germantown, MD, USA). Saliva samples were handled similarly, except that no homogenization was performed and 400 μL of media was used instead of 1 mL for suspension of the viral sample. Viral RNA was extracted using QIAamp^®^ Viral RNA Mini Kits from 140 µL samples. The Superscript III One-Step qRT-PCR with Platinum^®^ Taq Kit by Invitrogen (Invitrogen, Carlsbad, CA, USA), following the manufacturer’s protocol, was used to prepare RNA-extracted samples for quantitative real-time polymerase chain reaction (qRT-PCR) methods. Primers and probes specific to the Asian lineage of ZIKV were used. Primers were designed to target the NS5 gene with the following sequences: forward primer, 5′-CTTCTTATCCACAGCCGTCTC-3′; reverse primer, 5′-CCAGGCTTCAACGTCGTTAT-3′: and probe 5′-/56-FAM/AGAAGGAGACGAGATGCGGTACAGG/3BHQ_1/-3′ (Integrated DNA Technologies, Coralville, IA, USA). qRT-PCR methods were used to analyze the presence and titer of ZIKV RNA in mosquito samples. The qRT-PCR program used was: 30 min at 50 °C, 2.0 min at 94 °C, 12 s at 94 °C, 1 min at 58 °C, and lastly repeated for 39 cycles. The cutoff for positive ZIKV samples was set at a Cq detection of 35 PCR cycles. 

### 4.5. Zika Virus Standard Curve

A standard curve methodology was used to estimate PFU equivalents per mL by a plaque assay from Cq from qRT-PCR. We used 10-fold serial dilutions of viral stock for the qRT-PCR quantification of viral load. Viral RNA was extracted from each dilution using a QIAamp Viral RNA Mini Kit. The qRT-PCR primers and probe, along with the program, are described in an earlier section. The same serial dilutions used for PCR were also used as the basis for a plaque assay. The plaque assay involved inoculating monolayers of Vero cells in 6-well plates with 140 μL of serial dilution ZIKV (3-fold replication per dilution), followed by a 1 h incubation at 37 °C and at a 5% CO2 atmosphere. Following incubation, each well received a 2 mL agarose overlay (0.7%) followed by incubation for six additional days. After incubation, media and agarose were removed, and the plates were stained with crystal violet, rinsed with tap water, and visual plaques were counted.

### 4.6. Statistical Analysis

Treatment effects of mosquito species, time, and species-by-time interaction effects on infection responses were analyzed using logistic regression analysis (PROC LOGISTIC, SAS 9.4) based on the number of mosquitoes categorized for the presence or absence of ZIKV RNA. Separate analyses were performed to characterize susceptibility to infection (bodies), disseminated infection (legs), and transmission potential (saliva). Significant treatment effects were further examined using a pair-wise comparison follow-up test, correcting for multiple comparisons by the Tukey-Kramer method. The extrinsic incubation period (EIP_50_) was calculated as the time in days from imbibing ZIKV-infected blood until 50% of mosquitoes tested positive for ZIKV RNA in their saliva. A probit analysis (PROC PROBIT, SAS 9.4) was used to estimate EIP_50_ values. Analysis of variance (PROC GLM, SAS 9.4) was used to test for differences in virus titers in the body, legs, and saliva expectorates of infected mosquitoes. Significant effects were followed by pairwise comparisons using Tukey–Kramer multiple comparisons among least-squares means. Viral load in saliva expectorates approximates the inoculating dose delivered to a vertebrate host during biting.

## Figures and Tables

**Table 1:** Logistic regression analyses for species, time, and their interaction on susceptibility to infection, disseminated infection, and transmission potential for Zika virus. The species by time interaction is represented by Species*Time.

Mosquito Sample	Factor	DF	χ^2^	p-Value
Susceptibility to infection (body)	Species	1	3.26	0.0710
	Time	7	4.21	0.7555
	Species*Time	7	2.12	0.9527
Disseminated infection (legs)	Species	1	2.49	0.1145
	Time	7	70.43	<0.0001
	Species*Time	7	10.62	0.1558
Transmission potential (saliva)	Species	1	0.03	0.8523
	Time	7	19.98	0.0056
	Species*Time	7	14.59	0.0416

**Table 2:** Logistic regression of mosquito species and time (day) on susceptibility to infection (body). Results show the means (probability scale), standard errors, and 95% confidence intervals (lower and upper means) for susceptibility to infection.

Species	Time (Day)	Mean (No. Samples)	Std Error of Mean	Lower Mean	Upper Mean
*Ae. aegypti*	3	0.6875 (46)	0.06690	0.5444	0.8020
*Ae. aegypti*	6	0.7097 (60)	0.05765	0.5855	0.8088
*Ae. aegypti*	9	0.7647 (49)	0.05940	0.6299	0.8612
*Ae. aegypti*	12	0.7600 (48)	0.06040	0.6233	0.8584
*Ae. aegypti*	15	0.7857 (40)	0.06331	0.6370	0.8846
*Ae. aegypti*	18	0.7500 (46)	0.0625	0.6095	0.8522
*Ae. aegypti*	21	0.8605 (41)	0.05284	0.7224	0.9359
*Ae. aegypti*	24	0.7419 (29)	0.07859	0.5626	0.8654
*Ae. albopictus*	3	0.8182 (9)	0.1163	0.4930	0.9542
*Ae. albopictus*	6	0.9167 (10)	0.07979	0.5868	0.9884
*Ae. albopictus*	9	0.8000 (13)	0.1033	0.5302	0.9341
*Ae. albopictus*	12	0.7500 (10)	0.1250	0.4482	0.9172
*Ae. albopictus*	15	0.8333 (10)	0.1076	0.5228	0.9580
*Ae. albopictus*	18	0.7778 (7)	0.1386	0.4210	0.9440
*Ae. albopictus*	21	0.9444 (16)	0.05399	0.6935	0.9922
*Ae. albopictus*	24	0.8571 (12)	0.09352	0.5732	0.9640

**Table 3:** Logistic regression of mosquito species and time (day) on disseminated infection (legs). Results show means (probability scale), standard errors, and 95% confidence intervals (lower and upper means) for disseminated infection.

Species	Time (Day)	Mean (No. Samples)	Std Error of Mean	Lower Mean	Upper Mean
*Ae. aegypti*	3	0.0416 (46)	0.0288	0.0104	0.1519
*Ae. aegypti*	6	0.2742 (60)	0.0566	0.1778	0.3976
*Ae. aegypti*	9	0.4706 (49)	0.0698	0.3390	0.6064
*Ae. aegypti*	12	0.5800 (48)	0.0698	0.4406	0.7077
*Ae. aegypti*	15	0.7714 (40)	0.0709	0.6053	0.8814
*Ae. aegypti*	18	0.8649 (46)	0.0562	0.7138	0.9426
*Ae. aegypti*	21	0.8684 (41)	0.0548	0.7204	0.9442
*Ae. aegypti*	24	0.8261 (29)	0.0790	0.6177	0.9332
*Ae. albopictus*	3	0.0909 (9)	0.0867	0.0126	0.4386
*Ae. albopictus*	6	0.2500 (10)	0.1250	0.0827	0.5518
*Ae. albopictus*	9	0.1333 (13)	0.0877	0.0335	0.4054
*Ae. albopictus*	12	0.7000 (10)	0.1449	0.3763	0.9002
*Ae. albopictus*	15	0.5000 (10)	0.1443	0.2439	0.7561
*Ae. albopictus*	18	0.4286 (7)	0.1870	0.1437	0.7702
*Ae. albopictus*	21	0.8333 (16)	0.0878	0.5914	0.9454
*Ae. albopictus*	24	0.8571 (12)	0.0935	0.5732	0.9640

**Table 4:** Logistic regression of mosquito species and time (day) on potential transmission (saliva). Results show means (probability scale), standard errors, and 95% confidence intervals (lower and upper means) for saliva infection.

Species	Time (Day)	Mean (No. Samples)	Std Error of Mean	Lower Mean	Upper Mean
*Ae. aegypti*	3	0.0213 (46)	0.0210	0.0029	0.1362
*Ae. aegypti*	6	0.0483 (60)	0.0272	0.0156	0.1396
*Ae. aegypti*	9	0.1961 (49)	0.0556	0.1089	0.3275
*Ae. aegypti*	12	0.3000 (48)	0.0648	0.1897	0.4397
*Ae. aegypti*	15	0.3095 (40)	0.0713	0.1890	0.4630
*Ae. aegypti*	18	0.4167 (46)	0.0711	0.2869	0.5591
*Ae. aegypti*	21	0.4419 (41)	0.0757	0.3025	0.5910
*Ae. aegypti*	24	0.1935 (29)	0.0709	0.0896	0.3691
*Ae. albopictus*	3	0.0909 (9)	0.0867	0.0126	0.4386
*Ae. albopictus*	6	0.1667 (10)	0.1076	0.0419	0.4772
*Ae. albopictus*	9	0.1333 (13)	0.0877	0.0335	0.4054
*Ae. albopictus*	12	0.1667 (10)	0.1076	0.0419	0.4772
*Ae. albopictus*	15	0.5000 (10)	0.1443	0.2439	0.7561
*Ae. albopictus*	18	0.1112 (7)	0.1048	0.0154	0.4999
*Ae. albopictus*	21	0.1667 (16)	0.0878	0.0547	0.4086
*Ae. albopictus*	24	0.4286 (12)	0.1323	0.2065	0.6837

**Table 5:** Probit procedure by species was run to determine the probability values for the EIP. The median extrinsic incubation period (EIP_50_) was calculated as the time from ingestion of the infectious blood meal until 50% of infected females were capable of transmission. The estimated EIP_50_ values of *Ae. albopictus* are approximately 16 dpi and 18 dpi for *Ae. aegypti*.

	*Ae. aegypti*			*Ae. albopictus*		
Probability	dpi	95% Fiducial Limits		dpi	95% Fiducial Limits	
0.10	1.58	3.08	4.612	50.06	28.51	50.73
0.15	2.52	0.00001	5.96	40.36	25.19	95.85
0.20	3.66	0.0002	7.35	34.02	22.68	41.14
0.25	5.04	0.0029	8.85	29.37	20.56	27.86
0.30	6.70	0.02	10.57	25.75	18.56	25.18
0.35	8.74	0.21	12.74	22.79	16.40	27.94
0.40	11.24	1.43	16.29	20.29	13.55	37.33
0.45	14.33	6.48	28.47	18.14	8.61	69.63
0.50	18.21	12.39	113.53	16.24	2.57	28.56

**Table 6:** Analysis of variance (ANOVA) was used to test for viral load (log_10_ plaque-forming unit equivalents per mL) in bodies of infected *Ae. aegypti* and *Ae. albopictus* species to display the distribution of infection by species and time (dpi).

*Ae. aegypti*				*Ae. albopictus*		
dpi	*n*	Mean (Log_10_)	Std dev	*n*	Mean (Log_10_)	Std dev
3	36	2.78	0.87	8	2.70	0.54
6	43	4.32	0.71	10	4.14	1.22
9	38	4.77	0.76	11	4.89	1.05
12	36	5.67	0.33	8	5.70	0.25
15	32	5.32	0.82	9	5.50	0.54
18	35	5.23	1.24	6	4.40	1.65
21	36	5.12	1.28	16	5.73	0.36
24	22	5.15	0.95	11	5.35	1.31

**Table 7:** Analysis of variance (ANOVA) was used to test for viral load (log_10_ plaque-forming units per mL) in legs (disseminated infection) of infected mosquitoes by species (*Ae. aegypti* and *Ae. albopictus*) to display the distribution of disseminated infection by species and time (dpi).

*Ae. aegypti*				*Ae. albopictus*		
dpi	*n*	Mean (Log_10_)	Std dev	*n*	Mean (Log_10_)	Std dev
3	1	3.56	-	-	-	-
6	16	2.97	3.15	1	1.71	-
9	23	2.65	0.96	1	4.00	-
12	21	3.87	1.00	1	1.98	-
15	27	3.98	1.21	5	3.90	0.61
18	31	4.13	0.71	3	3.10	1.60
21	32	4.63	0.59	14	4.01	0.91
24	19	4.40	0.67	11	3.87	1.34

**Table 8:** Analysis of variance (ANOVA) was used to test for viral load (log_10_ plaque-forming units per mL) in saliva expectorates of infected *Ae. aegypti* and *Ae. albopictus* mosquitoes by species to display the distribution of infected saliva viral titer by species by time (dpi) interaction. There were no positive saliva samples to test for *Ae. aegypti* and *Ae. albopictus* at 3 dpi.

*Ae. aegypti*				*Ae. albopictus*		
dpi	*n*	Mean (Log_10_)	Std dev	*n*	Mean (Log_10_)	Std dev
3	-	-	-	-	-	-
6	2	3.62	3.71	1	1.17	-
9	9	3.24	3.04	1	2.05	-
12	14	2.49	0.83	1	1.68	-
15	12	2.69	0.93	5	3.92	4.06
18	19	2.28	0.81	-	-	-
21	18	1.92	0.62	2	1.50	0.29
24	5	2.28	0.97	5	2.49	1.22
